# Supplementary material for: Dual-Wavelength Excited Intense Red Upconversion Luminescence from Er3+-Sensitized Y2O3 Nanocrystals Fabricated by Spray Flame Synthesis
Source: Nanomaterials (Basel). 2020 Jul 28;10(8):1475. doi: 10.3390/nano10081475 (PMC7466467; doi:10.3390/nano10081475)
Supplement: Supplementary file 1 [file nanomaterials-10-01475-s001.pdf]

nanomaterials

Supplementary Materials

Dual-Wavelength Excited Intense Red Upconversion Luminescence from Er^{3+}-Sensitized Y_2O_3 Nanocrystals Fabricated by Spray Flame Synthesis

Xiaofan Zhao [1,2,†], Zeyun Wu [3,†], Zining Yang [1,2,†], Xu Yang [1,2], Yiyang Zhang [4], Maohui Yuan [1,5,*], Kai Han [1,2], Changqing Song [1,2], Zongfu Jiang [1,2], Hongyan Wang [1,2,*], Shuiqing Li [3,*] and Xiaojun Xu [1,2,6]

[1] College of Advanced Interdisciplinary Studies, National University of Defense Technology, Changsha 410073, China; zhaoxiaofan11@nudt.edu.cn (X.Z.); diablo_3rd@126.com (Z.Y.); fractal_yangxu@outlook.com (X.Y.); hankai0071@nudt.edu.cn (K.H.); songchangqing08@nudt.edu.cn (C.S.); jiangzongfu7@163.com (Z.J.); xuxj@21.cn.com (X.X.)
[2] Hunan Provincial Key Laboratory of High Energy Laser Technology, National University of Defense Technology, Changsha 410073, China
[3] Department of Energy and Power Engineering, Key Laboratory for Thermal Science and Power Engineering of Ministry of Education, Tsinghua University, Beijing 100084, China; wuzeyun18@mails.tsinghua.edu.cn
[4] Key Laboratory of Advanced Reactor Engineering and Safety of Ministry of Education, Collaborative Innovation Center of Advanced Nuclear Energy Technology, Institute of Nuclear and New Energy Technology, Tsinghua University, Beijing 100084, China; zhangyiyang@mail.tsinghua.edu.cn
[5] Department of Physics and Chemistry, PLA Army Academy of Special Operations, Guangzhou 510507, China
[6] State Key Laboratory of Pulsed Power Laser Technology, National University of Defense Technology, Changsha 410073, China
* Correspondence: yuanmaohuino1@126.com (M.Y.); wanghongyan@nudt.edu.cn (H.W.); lishuiqing@tsinghua.edu.cn (S.L.)
† These authors contributed equally to this work.

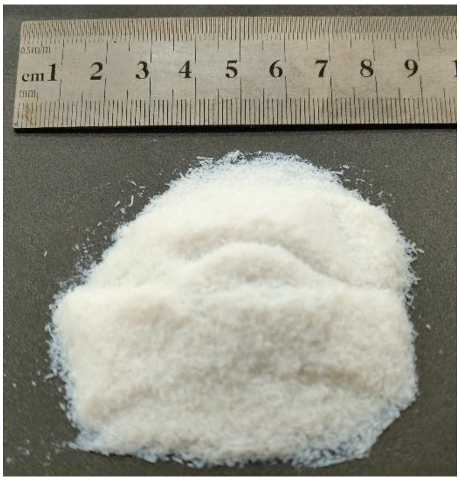

Figure S1. The synthesized Y_2O_3:Er^{3+}/Tm^{3+} UCNPs.

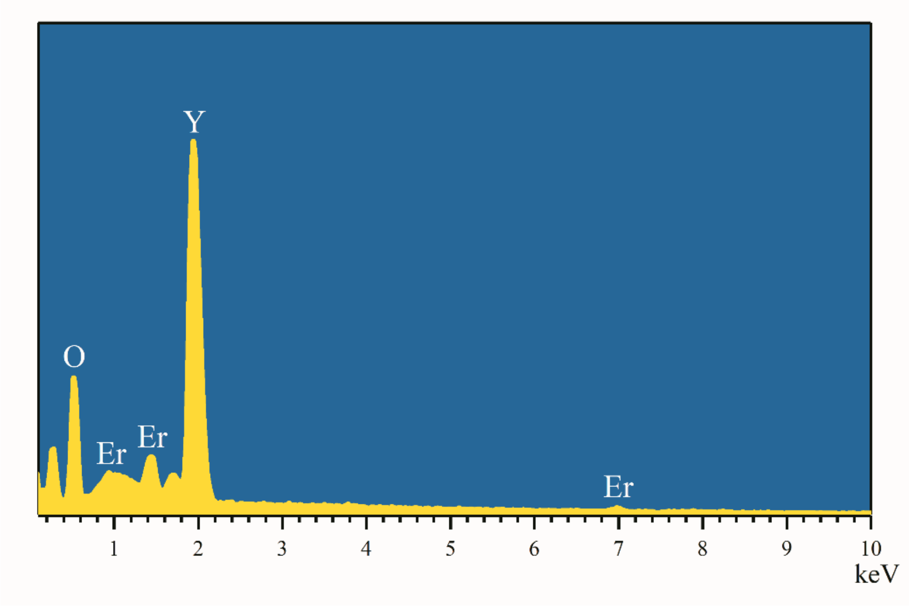

Figure S2. The EDS spectra of the synthesized Y_2O_3 nanoparticles doped with 8 mol% Er^{3+} ions.

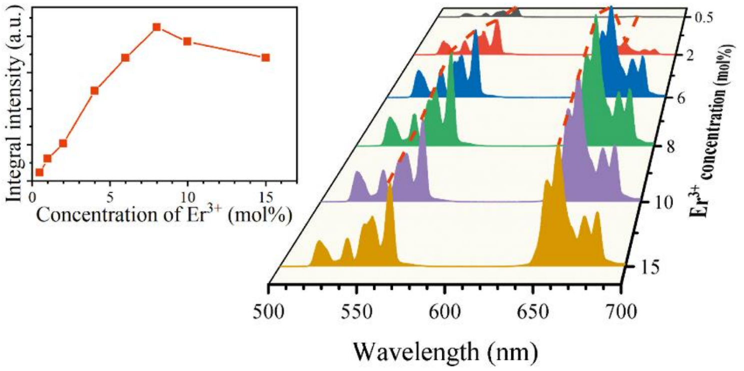

Figure S3. The absolute luminescence spectra of the Y_2O_3:Er^{3+} UCNPs doped with different concentration of Er^{3+} ions. The inset shows the tendency of the integral luminescence intensities (from 500 to 700 nm). All excitation wavelengths are at 980 nm.

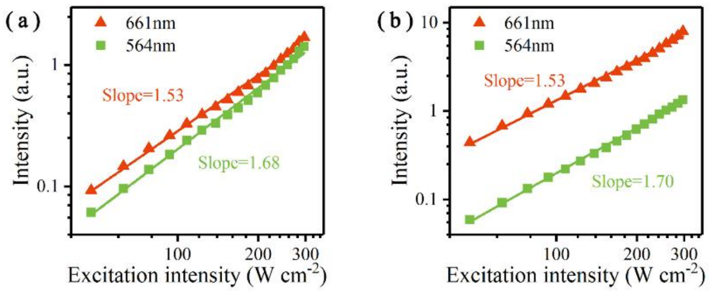

Figure S4. UC emission intensities of Y_2O_3:Er^{3+}/Tm^{3+} (8/*x* mol%) UCNPs as a function of excitation intensities. (**a**) $x = 0$, (**b**) $x = 1$. All excitation wavelengths are at 808 nm.

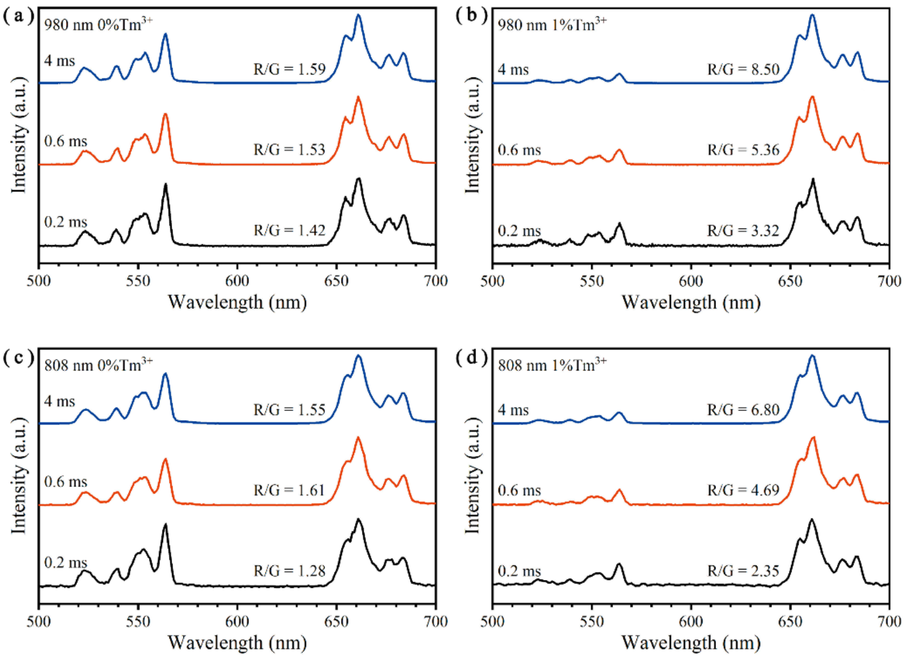

Figure S5. Normalized UC emission spectra of Y_2O_3: Er^{3+}/Tm^{3+} (8/*x* mol%) UCNPs under the excitation of lasers operated at different pulse width. (a) $x = 0$, 980 nm excitation, (b) $x = 1$, 980 nm excitation, (c) $x = 0$, 808 nm excitation, (d) $x = 1$, 808 nm excitation.

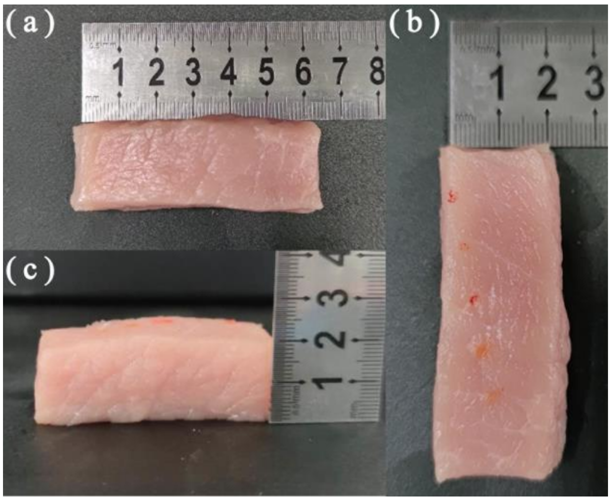

Figure S6. Cuboid fresh pork with the size of 6 cm × 2 cm × 2 cm.

Table S1. The experimental elemental composition of the Y_2O_3:Er^{3+} (8 mol%) UCNPs.

Elements	Weight percent (%)	Atom percent (%)
O	31.69	73.74
Y	56.32	23.59
Er	11.99	2.67
Total	100.00	100.00

© 2020 by the authors. Submitted for possible open access publication under the terms and conditions of the Creative Commons Attribution (CC BY) license (http://creativecommons.org/licenses/by/4.0/).
